# Lower social vulnerability is associated with a higher prevalence of social media-involved violent crimes in Prince George’s County, Maryland, 2018–2023

**DOI:** 10.1186/s40621-024-00538-w

**Published:** 2024-09-30

**Authors:** Jemar R. Bather, Diana Silver, Brendan P. Gill, Adrian Harris, Jin Yung Bae, Nina S. Parikh, Melody S. Goodman

**Affiliations:** 1 Center for Anti-racism, Social Justice & Public Health, New York University School of Global Public Health, 708 Broadway 9th Floor, New York, NY 10003 USA; 2 Department of Biostatistics, New York University School of Global Public Health, New York, NY 10003 USA; 3 Department of Public Health Policy and Management, New York University School of Global Public Health, New York, NY 10003 USA; 4 Prince George’s County Police Department, Upper Marlboro, MD 20774 USA; 5 Department of Social and Behavioral Sciences, New York University School of Global Public Health, New York, NY 10003 USA

**Keywords:** Internet, Place, Injury, Health inequities, Urban, Stratification, Social class, Weapon, Firearms, Safety

## Abstract

**Background:**

Social vulnerability may play a role in social media-involved crime, but few studies have investigated this issue. We investigated associations between social vulnerability and social media-involved violent crimes.

**Methods:**

We analyzed 22,801 violent crimes occurring between 2018 and 2023 in Prince George’s County, Maryland. Social media involvement was obtained from crime reports at the Prince George’s County Police Department. Social media application types included social networking, advertising/selling, ridesharing, dating, image/video hosting, mobile payment, instant messaging/Voice over Internet Protocol, and other. We used the Centers for Disease Control and Prevention’s Social Vulnerability Index to assess socioeconomic status (SES), household characteristics, racial and ethnic minority status, housing type and transportation, and overall vulnerability. Modified Poisson models estimated adjusted prevalence ratios (aPRs) among the overall sample and stratified by crime type (assault and homicide, robbery, and sexual offense). Covariates included year and crime type.

**Results:**

Relative to high tertile areas, we observed a higher prevalence of social media-involved violent crimes in areas with low SES vulnerability (aPR: 1.82, 95% CI: 1.37-2.43), low housing type and transportation vulnerability (aPR: 1.53, 95% CI: 1.17-2.02), and low overall vulnerability (aPR: 1.63, 95% CI: 1.23-2.17). Low SES vulnerability areas were significantly associated with higher prevalences of social media-involved assaults and homicides (aPR: 1.64, 95% CI: 1.02-2.62), robberies (aPR: 2.00, 95% CI: 1.28-3.12), and sexual offenses (aPR: 2.07, 95% CI: 1.02-4.19) compared to high SES vulnerability areas. Low housing type and transportation vulnerability (vs. high) was significantly associated with a higher prevalence of social media-involved robberies (aPR: 1.54, 95% CI:1.01-2.37). Modified Poisson models also indicated that low overall vulnerability areas had higher prevalences of social media-involved robberies (aPR: 1.71, 95% CI: 1.10-2.67) and sexual offenses (aPR: 2.14, 95% CI: 1.05-4.39) than high overall vulnerability areas.

**Conclusions:**

We quantified the prevalence of social media-involved violent crimes across social vulnerability levels. These insights underscore the need for collecting incident-based social media involvement in crime reports among law enforcement agencies across the United States and internationally. Comprehensive data collection at the national and international levels provides the capacity to elucidate the relationships between neighborhoods, social media, and population health.

**Supplementary Information:**

The online version contains supplementary material available at 10.1186/s40621-024-00538-w.

## Background

Social media is increasingly important in everyday life (Fernández-Planells et al. 2021; Hyatt et al. 2021). It can be defined as “any online resource that is designed to facilitate engagement between individuals (Aichner et al. 2021; Bishop 2019).” Data from the Pew Research Center show that the percentage of Americans using at least one social media platform exponentially grew from 5% in 2005 to 72% in 2011 (Pew Research Center 2021). Reasons for social media usage often include content sharing, entertainment purposes, and news coverage (Pew Research Center 2021). Emerging studies from the social sciences suggest that social media is also used in preparation and perpetuation of crime in the US and beyond (Décary-Hétu and Morselli 2011; Lane 2016; Lauger et al. 2020; Patton et al. 2014, 2014, 2016). The digital landscape of social media platforms allows gang members to instantly share messages, images, and videos to expand their membership, threaten rival gangs, and sell illicit drugs (Moule et al. 2014; Patton et al. 2013, 2017, 2019; Peterson and Densley 2017; Pyrooz et al. 2015; Storrod and Densley 2017; Stuart 2020). Consequently, these actions lead to hostile environments manifested through in-person conflict and offline retaliation (Lane 2016; Patton et al. 2013, 2018). In addition, the posting of one’s whereabouts or activities may also provide valuable information about a potential victim’s whereabouts to those seeking to commit violent or property crimes.

Understanding social media-involved crimes is crucial because it allows researchers and law enforcement agencies to identify new criminal activity patterns and methods, develop targeted prevention strategies, and better allocate resources to combat emerging threats in the digital age (Moore and Stuart 2022). This understanding can also inform public policy decisions regarding online safety and social media regulation. The Prince George’s County Police Department in Maryland has advanced the field by being the sole US law enforcement agency to systematically track social media’s role at the incident-based level of criminal activities (Garcia Whitlock et al. 2023). Officers from this police department note on the crime report whether social media was involved in the crime and indicate the specific social media platform used (e.g., Instagram, Facebook, TikTok). A recent study analyzing these data showed that crimes involving social media had a higher percentage of robberies (48% vs. 20%) and carjackings (12% vs. 5%) than those without social media involvement (Garcia Whitlock et al. 2023). We build on this work by investigating whether neighborhood characteristics, especially those that show evidence of residential structural racism, increase the likelihood of social media-involved crimes.

A substantial body of work has documented the role of structural racism in creating inequities across neighborhoods with minoritized populations (Bailey et al. 2021; LaVeist et al. 2023). Systemic inequities disproportionally and adversely affect resource-poor communities resulting in disparate levels of socially vulnerability (Amaro et al. 2021; Deziel et al. 2023; Givens et al. 2021; King et al. 2022; Ryan et al. 2024). Discriminatory practices like redlining and residential segregation have shown to be associated with racial and ethnic minorities’ reduced healthcare access and utilization, increased exposure to environmental toxins and chemicals, and limited access to healthy foods (Bullard 2000; Williams et al. 2019). Robust data links these structural conditions to racially and ethnically minoritized individuals experiencing higher rates of chronic disease and mortality than their White counterparts (Bailey et al. 2017; Williams et al. 2019). Relatedly, a well-documented relationship exists between highly disadvantaged neighborhoods and increased crime rates, leading to high proportions of minoritized individuals being policed, arrested, convicted, and incarcerated (Arnio 2021; Beck 2019; Braga et al. 2019; Gelman et al. 2007; Johnson et al. 2019; Schwartz and Jahn 2020; Siegel et al. 2019; Western 2006; Zare et al. 2022). Growing up in an underprivileged community increases the likelihood of getting initiated into a gang, becoming a homicide victim, and gaining access to firearms (Lane 2016; Merrin et al. 2015; Patton et al. 2013; Roberto et al. 2018; Santilli et al. 2017).

What is less clear is how social media crimes intersect with geographical segregation by race. Extant literature has shown that gangs in racially minoritized neighborhoods increasingly incorporate social media into their activities (Décary-Hétu and Morselli 2011; Moule et al. 2014; Peterson and Densley 2017; Pyrooz et al. 2015). However, does that mean social media is exacerbating crime rates in these resource-poor, minoritized communities? Or does social media simply make the already-existing crime prevalence more visible? Or does it lead to a new crime trend altogether? Much of the literature has focused on the role of social media as a tool for law enforcement, with some considering it to be new opportunities to solve crimes and others raising concern about it perpetuating negative racial stereotypes and disproportionately being used to target minorities (Aghababaei and Makrehchi 2016; Lane et al. 2018; Malleson and Andresen 2015). However, few examined the relationship between social media proliferation and crime prevalence in minority communities with high social vulnerability. Does social media make them more vulnerable to crime or not? This study aims to fill the gap in literature.

Identifying structurally driven mechanisms of social media-involved crimes holds important implications for social workers, prevention scientists, injury epidemiologists, and police departments. With this understanding, social workers can develop targeted outreach programs addressing digital safety for vulnerable communities; prevention scientists can design tailored social-media involved crime prevention strategies; injury epidemiologists could refine their surveillance methods to include social media factors in violence risk assessments; and police departments may adjust their resource allocation and community engagement strategies based on neighborhood-specific social media crime patterns.

To conduct this study, we leveraged crime reports from a unique data source to investigate associations between social vulnerability and social media-involved violent crimes in Prince George’s County, Maryland, from 2018 to 2023. Past studies and surveys have shown that the burden of violent crimes is disproportionate across racial and ethnic groups and that racial minorities generally tend to use social media more frequently than their white counterparts (Orcés, 2020; Zimmerman et al. 2024). In addition, while it is true that social media utilization rate tends to be higher among higher-income and more highly educated populations, social media is still widely utilized among even the lowest income and the least educated groups, with the vast majority reporting use of at least one main social media platforms (Gottfried, 2024). We thus hypothesized that, minoritized neighborhoods, especially those with greater social vulnerability, would be associated with a higher prevalence of social media-involved crime. Empirical insights from this study can inform violence prevention strategies, designing interventions, and crafting policies at the intersection of place, social media, and violent crime.

## Methods

### Study design and setting

We obtained crime records from the Prince George’s County Police Department in Maryland. These case reports included crimes that occurred between January 1, 2018, to December 31, 2023. The crime records included details such as where the incident occurred, an incident-based level indicator of whether social media was involved, and victim characteristics. The Prince George’s County Police Department is the fourth-largest law enforcement agency in Maryland (Prince George’s County 2023), with 2,100 law enforcement officials serving an area of nearly 950,000 residents who predominantly identify as Black or African American (64%), followed by White (27%). Census estimates indicate that the median household income in Prince George’s County is $97,935, 87% of individuals aged 25 or older have at least a high school diploma, and 11% live in poverty (US Census Bureau 2024). The median household income in Maryland is $98,461, with 91% of individuals aged 25 or older having at least a high school diploma, and 10% living in poverty (US Census Bureau 2024). This study adhered to the Declaration of Helsinki and the Strengthening the Reporting of Observational Studies in Epidemiology guidelines (von Elm et al. 2007). The New York University Institutional Review Board considered this secondary data analysis exempt from review.

## Analytic sample

The Prince George’s County Police Department first implemented social media data collection in 2017. The first full year of social media-involved data became reliable enough for academic use in 2018. The 2017 data were not complete and excluded for implementation, data quality, consistency, and training reasons. This approach aligns with the general practice for academic research partnerships with the Prince George’s County Police Department.

**Fig. 1:**
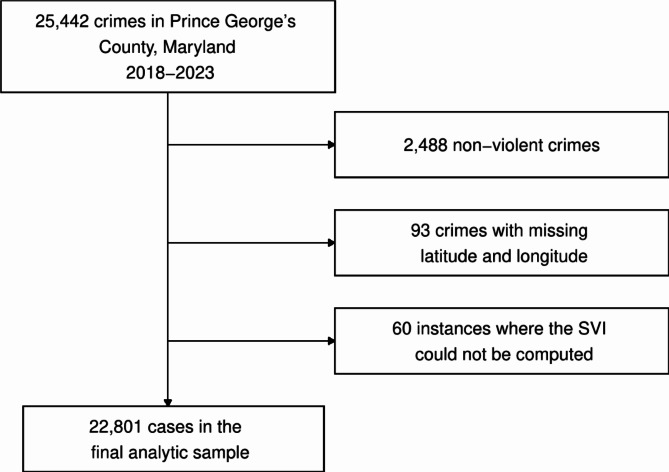
Analytic Sample Diagram, Social Media-Involved Violent Crimes in Prince George’s County, Maryland, 2018–2023

Figure 1 shows the analytic sample derivation. A total of 25,442 crimes occurred in Prince George’s County, Maryland, between 2018 and 2023. We excluded 2,488 (9.8%) non-violent crimes, 93 (0.4%) cases with missing latitude and longitudinal coordinates, and 60 (0.2%) instances where the Social Vulnerability Index could not be computed. A list of non-violent crimes is shown in Supplemental Table 1. Examples of non-violent crimes included motor vehicle theft, breaking and entering, driving under the influence, and fraud. The final analytic sample included 22,801 violent crimes. Violent crimes included simple and aggravated assault, robbery, carjacking, homicide (murder and manslaughter), sexual offense (rape, sexual assault with an object, and sodomy), and kidnapping/abduction (Supplemental Table 1).

## Social media-involved violent crime

The Prince George’s County Police Department required all officers (including non-Prince George’s County Police Department officers employed by any of the 33 municipalities) to indicate whether the reported incident had any nexus to a social media platform. The PremierOne Records Management System prompted officers with the following question: “Does this incident have any social media involvement?” An officer obtained an answer either by asking a reporting person (e.g., victim or witness) or by investigative leads (e.g., search and seizure warrants). When an officer selected “yes”, indicating that an incident does have a social media nexus, a multi-select field appeared. The officer’s screen listed the most common social media programs/applications. This was a multi-select option allowing the officer to choose one or multiple options, including the name of the social media site (e.g., OK Cupid, Wallapop, Kik, Scruff), how involved parties used the denoted application(s), and if there were any user/screen names. The example in the Prince George’s County Police Department Report Writing Manual is as follows: *“2A1 responds to an address for a citizen robbery. Once on the scene*,* the victim informs 2A1 that they were using “LetGo” to sell an iPhone. Once at the location of the sale*,* the suspects robbed the victim.”* This would be an example of a social media-involved robbery, and LetGo would be the social media application. In summary, if a person used an electronic digital network to communicate with another person for any reason (except for traditional communication methods such as voice call, e-mail, or text/SMS message), the Prince George’s County Police Department considered it a social media application. The primary outcome of interest was a dichotomous measure of whether social media was involved in the violent crime. Social media application types included social networking (Facebook, Instagram, Snapchat, TikTok, Twitter), advertising/selling (Backpage.com, Craigslist, LetGo, OfferUp, 5Mile), ridesharing (Uber, Lyft), dating (Grindr, Match.com, Plenty of Fish, Skout, Tinder), image/video hosting (YouTube, Flickr), mobile payment (CashApp, Zelle), instant messaging/Voice over Internet Protocol (Discord, Skype, WhatsApp), and Other (e.g., air drop/file share).

## Social vulnerability

We assessed social vulnerability using the Centers for Disease Control and Prevention’s Social Vulnerability Index (Centers for Disease Control and Prevention 2024). This index measures a census tract’s social vulnerability according to 16 criteria (Table 1). Each criterion was based on census variables from the 2017–2021 American Community Survey 5-year estimates (Centers for Disease Control and Prevention 2024). These criteria are categorized into four themes: socioeconomic status (SES), household characteristics, racial and ethnic minority status, and housing type and transportation. For the analyses, we examined each theme separately and as a sum indicating an area’s overall social vulnerability. We obtained census tract-level social vulnerability measures using latitude and longitudinal coordinates from the crime records. Social Vulnerability Index values range from 0 to 1, with higher scores reflecting greater social vulnerability (Centers for Disease Control and Prevention 2024). For example, an overall social vulnerability score of 0.44 indicates that the area ranks at the 44th percentile. We categorized these scores into low, medium, and high groups. Evaluating social vulnerability at the census tract level aligns with recommendations from the Public Health Disparities Geocoding Project (Testa et al. 2022).

**Table 1:** Social Vulnerability Index based on 2017–2021 American Community Survey 5-year estimates, Centers for Disease Control and Prevention

Composite measure	Theme	Census variables
Overall Social Vulnerability	Socioeconomic Status	• Below 150% Poverty • Unemployed • Housing Cost Burden • No High School Diploma
	Household Characteristics	•$\:\ge\:$65 years old •$\:\le\:$17 years old • Civilian with a Disability • Single-Parent Households • English Language Proficiency
	Racial & Ethnic Minority Status*	• Hispanic or Latino (of any race) • Black or African American, Not Hispanic or Latino • Asian, Not Hispanic or Latino • American Indian or Alaska Native, Not Hispanic or Latino • Native Hawaiian or Pacific Islander, Not Hispanic or Latino • Two or More Races, Not Hispanic or Latino • Other Races, Not Hispanic or Latino
	Housing Type & Transportation	• Multi-Unit Structures • Mobile Homes • Crowding • No Vehicle • Group Quarters

*Racial & Ethnic Minority Status census variables are counted as one social factor

## Victim and crime information

We obtained victim (age, sex, ethnicity, race, resident status) and crime information (type, drug-related, year) from crime reports. Since some cases had a range reported for age (e.g., 18–21, 20–30, 20–40), we recategorized age as < 18, 18–45, > 45 or missing. Sex was measured in the crime reports as male, female, or unknown. We collapsed unknown sex with missing sex due to small sample sizes. Ethnicity was categorized as non-Hispanic, Hispanic, or unknown/missing. Race was measured as White, Black, Asian, American Indian/Alaska Native, Native Hawaiian/Other Pacific Islander, or unknown/missing. Resident status of where the crime occurred was measured as resident, non-resident, or unknown/missing. Crime type was coded as simple assault, aggravated assault, robbery, carjacking, homicide, sexual offense, or kidnapping/abduction. Drug-related crime was classified as yes, no, or missing.

### Statistical analysis

We tabulated descriptive statistics for all variables. Categorical measures were expressed as counts and percentages. We used Pearson’s Chi-squared test to perform bivariate analyses (Goodman 2017). We employed modified Poisson regression to analyze the adjusted associations between social vulnerability measures and social media-involved violent crimes (Zou 2004; Zou and Donner 2013). This method is preferable for estimating prevalence ratios (PRs) with binary data, offering better convergence than log-binomial regression and correcting for inflated standard errors produced by standard Poisson regression (Yelland et al. 2011; Zou 2004). We implemented modified Poisson regression models using generalized estimating equations, incorporating an exchangeable correlation structure to account for dependence within census tract (Fitzmaurice et al. 2011; Liang and Zeger 1986). To ensure robustness, we conducted a sensitivity analysis comparing the modified Poisson model estimates to those obtained from negative binomial regression models. All statistical analyses were conducted in Stata/SE 18.0 (StataCorp 2023).

## Results

### Sample characteristics

Among the analytic sample of 22,801 violent crimes in Prince George’s County, 200 census tracts were represented, with an average of 114 crime reports per census tract. Most victims were between the ages of 18 and 45 (67%), non-Hispanic (57%), Black (66%), and residents of where the crime occurred (75%, Table 2). 50% of the victims were male, 42% of the violent crimes were simple assaults, 98% were not drug related, and 19% occurred in 2023. About 40% of the cases happened in high social vulnerability areas: SES (41% in the 67th − 100th percentile group), household characteristics (39% in the 68th − 100th percentile group), racial and ethnic minority status (55% in the 66th − 98th percentile group), housing type and transportation (41% in the 67th − 100th percentile group), and overall (47% in the 66th − 100th percentile group, Table 3).

**Table 2:** Analytic sample characteristics, overall and by social media-involved status, violent crimes in Prince George’s County, Maryland, 2018–2023

	Overall	Social media-involved violent crime		*P* value
		No	Yes	
	No. (%)	No. (%)	No. (%)	
	22,801 (100.0%)	22,381 (98.2%)	420 (1.8%)	
**Age**				< 0.001
<18	2,344 (10.3%)	2,259 (10.1%)	85 (20.2%)	
18–45	15,256 (66.9%)	14,964 (66.9%)	292 (69.5%)	
>45	3,852 (16.9%)	3,812 (17.0%)	40 (9.5%)	
Missing	1,349 (5.9%)	1,346 (6.0%)	3 (0.7%)	
**Sex**				< 0.001
Male	11,362 (49.8%)	11,105 (49.6%)	257 (61.2%)	
Female	10,095 (44.3%)	9,935 (44.4%)	160 (38.1%)	
Unknown/Missing	1,344 (5.9%)	1,341 (6.0%)	3 (0.7%)	
**Ethnicity**				0.12
non-Hispanic	12,995 (57.0%)	12,749 (57.0%)	246 (58.6%)	
Hispanic	4,728 (20.7%)	4,631 (20.7%)	97 (23.1%)	
Unknown/Missing	5,078 (22.3%)	5,001 (22.3%)	77 (18.3%)	
**Race**				< 0.001
White	5,783 (25.4%)	5,654 (25.3%)	129 (30.7%)	
Black	14,933 (65.5%)	14,673 (65.6%)	260 (61.9%)	
Asian	366 (1.6%)	350 (1.6%)	16 (3.8%)	
American Indian/Alaska Native	132 (0.6%)	126 (0.6%)	6 (1.4%)	
Native Hawaiian/Other Pacific Islander	109 (0.5%)	106 (0.5%)	3 (0.7%)	
Unknown/Missing	1,478 (6.5%)	1,472 (6.6%)	6 (1.4%)	
**Resident status**				< 0.001
Resident	17,038 (74.7%)	16,769 (74.9%)	269 (64.0%)	
Nonresident	3,329 (14.6%)	3,191 (14.3%)	138 (32.9%)	
Unknown/Missing	2,434 (10.7%)	2,421 (10.8%)	13 (3.1%)	
**Crime type**				< 0.001
Simple Assault	9,474 (41.6%)	9,403 (42.0%)	71 (16.9%)	
Aggravated Assault	5,294 (23.2%)	5,252 (23.5%)	42 (10.0%)	
Robbery	6,477 (28.4%)	6,246 (27.9%)	231 (55.0%)	
Sexual Offense	1,044 (4.6%)	977 (4.4%)	67 (16.0%)	
Homicide	460 (2.0%)	453 (2.0%)	7 (1.7%)	
Kidnapping/Abduction	52 (0.2%)	50 (0.2%)	2 (0.5%)	
**Drug-related crime**				0.66
No	22,235 (97.5%)	21,828 (97.5%)	407 (96.9%)	
Yes	535 (2.3%)	523 (2.3%)	12 (2.9%)	
Missing	31 (0.1%)	30 (0.1%)	1 (0.2%)	
**Year**				0.033
2018	3,943 (17.3%)	3,884 (17.4%)	59 (14.0%)	
2019	3,869 (17.0%)	3,806 (17.0%)	63 (15.0%)	
2020	3,612 (15.8%)	3,553 (15.9%)	59 (14.0%)	
2021	3,163 (13.9%)	3,100 (13.9%)	63 (15.0%)	
2022	3,836 (16.8%)	3,743 (16.7%)	93 (22.1%)	
2023	4,378 (19.2%)	4,295 (19.2%)	83 (19.8%)	

P value from a Pearson’s Chi-squared test

**Table 3:** Bivariate associations between social vulnerability and social media-involved status, violent crimes in Prince George’s County, Maryland, 2018–2023

	Overall	Social media-involved violent crime		*P* value
		No	Yes	
	No. (%)	No. (%)	No. (%)	
	22,801 (100.0%)	22,381 (98.2%)	420 (1.8%)	
**Social vulnerability theme**				
**Socioeconomic status**				< 0.001
Low (0.001,0.333]	3,625 (15.9%)	3,521 (15.7%)	104 (24.8%)	
Medium (0.333,0.667]	9,940 (43.6%)	9,769 (43.6%)	171 (40.7%)	
High (0.667,1.000]	9,236 (40.5%)	9,091 (40.6%)	145 (34.5%)	
**Household characteristics**				0.48
Low (0.001,0.333]	6,248 (27.4%)	6,130 (27.4%)	118 (28.1%)	
Medium (0.333,0.667]	7,667 (33.6%)	7,517 (33.6%)	150 (35.7%)	
High (0.667,1.000]	8,886 (39.0%)	8,734 (39.0%)	152 (36.2%)	
**Racial & ethnic minority status**				0.74
Low (0.001,0.327]	3,074 (13.5%)	3,013 (13.5%)	61 (14.5%)	
Medium (0.327,0.654]	7,303 (32.0%)	7,166 (32.0%)	137 (32.6%)	
High (0.654,0.982]	12,424 (54.5%)	12,202 (54.5%)	222 (52.9%)	
**Housing type & transportation**				< 0.001
Low (0.001,0.333]	4,525 (19.8%)	4,409 (19.7%)	116 (27.6%)	
Medium (0.333,0.667]	8,917 (39.1%)	8,771 (39.2%)	146 (34.8%)	
High (0.667,1.000]	9,359 (41.0%)	9,201 (41.1%)	158 (37.6%)	
**Overall**				< 0.001
Low (0.001,0.333]	3,602 (15.8%)	3,508 (15.7%)	94 (22.4%)	
Medium (0.333,0.667]	8,409 (36.9%)	8,254 (36.9%)	155 (36.9%)	
High (0.667,1.000]	10,790 (47.3%)	10,619 (47.4%)	171 (40.7%)	

P value from a Pearson’s Chi-squared test

Of the 22,801 violent crimes, 420 (2%) had social media involvement (Table 4). The most common social media application types used in these crimes were social networking (42%) and advertising/selling (20%). Application type significantly differed by crime type (*P* < 0.001). Social networking applications were more likely to be used in assaults and homicides, advertising/selling applications were more likely to be used in robberies, and dating applications were more likely to be used in sexual offenses.

**Table 4:** Distribution of social media application type by violent crime type, violent crimes in Prince George’s County, Maryland, 2018–2023

	Overall	Crime type						*P* value
		Simple Assault	Aggravated Assault	Robbery	Sexual Offense	Homicide	Kidnapping/Abduction	
	No. (%)	No. (%)	No. (%)	No. (%)	No. (%)	No. (%)	No. (%)	
	420 (100.0%)	71 (16.9%)	42 (10.0%)	231 (55.0%)	67 (16.0%)	7 (1.7%)	2 (0.5%)	
**Application type**								<0.001
Social networking	178 (42.4%)	45 (63.4%)	27 (64.3%)	66 (28.6%)	34 (50.7%)	5 (71.4%)	1 (50.0%)	
Advertising/Selling	82 (19.5%)	0 (0.0%)	3 (7.1%)	78 (33.8%)	0 (0.0%)	1 (14.3%)	0 (0.0%)	
Ridesharing	55 (13.1%)	13 (18.3%)	7 (16.7%)	34 (14.7%)	0 (0.0%)	0 (0.0%)	1 (50.0%)	
Dating	21 (5.0%)	1 (1.4%)	0 (0.0%)	9 (3.9%)	11 (16.4%)	0 (0.0%)	0 (0.0%)	
Image/Video Hosting	2 (0.5%)	0 (0.0%)	0 (0.0%)	1 (0.4%)	1 (1.5%)	0 (0.0%)	0 (0.0%)	
Mobile payment	10 (2.4%)	1 (1.4%)	0 (0.0%)	7 (3.0%)	2 (3.0%)	0 (0.0%)	0 (0.0%)	
Instant messaging/Voice over Internet Protocol	2 (0.5%)	0 (0.0%)	0 (0.0%)	1 (0.4%)	1 (1.5%)	0 (0.0%)	0 (0.0%)	
Other	70 (16.7%)	11 (15.5%)	5 (11.9%)	35 (15.2%)	18 (26.9%)	1 (14.3%)	0 (0.0%)	

### Sample characteristics by social media-involved status

We observed statistically significant differences in several characteristics by social media involvement status (Tables 2 and 3). Compared to crimes not involving social media, social media-involved crimes had higher proportions of victims who were less than 18 years old (20% vs. 10%, *P* < 0.001), male (61% vs. 50%, *P* < 0.001), White (31% vs. 25%, *P* < 0.001), and non-residents of the area where the crime occurred (33% vs. 14%, *P* < 0.001, Table 2). Social media-involved violent crimes included a higher percentage of robberies than violent crimes not involving social media (55% vs. 28%, *P* < 0.001). Compared to crimes not involving social media, social media-involved crimes were more likely to occur in areas of low SES (25% vs. 16%, *P* < 0.001), housing type and transportation (28% vs. 20%, *P* < 0.001), and overall vulnerability (22% vs. 16%, *P* < 0.001, Table 3).

### Associations between social vulnerability and social media-involved violent crimes

We found statistically significant relationships between three social vulnerability metrics and the prevalence of social media-involved crimes (Table 5). Relative to areas in the high group, we observed a higher prevalence of social media-involved violent crimes in areas with low SES vulnerability (adjusted PR [aPR]: 1.82, 95% CI: 1.37–2.43), low housing type and transportation vulnerability (aPR: 1.53, 95% CI: 1.17–2.02), and low overall vulnerability (aPR: 1.63, 95% CI: 1.23–2.17). We observed variation in the magnitude and statistical significance of the association across stratified analyses by crime type. Low SES vulnerability areas were significantly associated with higher prevalences of social media-involved assaults and homicides (aPR: 1.64, 95% CI: 1.02–2.62), robberies (aPR: 2.00, 95% CI: 1.28–3.12), and sexual offenses (aPR: 2.07, 95% CI: 1.02–4.19) compared to high SES vulnerability areas. Low housing type and transportation vulnerability (vs. high) was significantly associated with a higher prevalence of social media-involved robberies (aPR: 1.54, 95% CI: 1.01–2.37), but not with a higher prevalence of social media-involved assaults and homicides (aPR: 1.51, 95% CI: 0.97–2.35) or sexual offenses (aPR: 1.54, 95% CI: 0.76–3.11). Modified Poisson models also indicated that low overall vulnerability areas had higher prevalences of social media-involved robberies (aPR: 1.71, 95% CI: 1.10–2.67) and sexual offenses (aPR: 2.14, 95% CI: 1.05–4.39) than high overall vulnerability areas. Sensitivity analyses based on negative binomial regression models yielded similar results (Supplemental Table 2). Models among all crime types controlled for crime type and incident year. Stratified models controlled for incident year. Modified Poisson and negative binomial regression models did not converge for stratified models based on kidnapping and abduction cases.

**Table 5:** Lower social vulnerability is associated with a higher prevalence of social media-involved violent crimes in Prince George’s County, Maryland, 2018–2023

	All crime types^a^		Crime type (subgroup analyses)^b^					
			Assault & homicide		Robbery		Sexual offense	
	PR	95% CI	PR	95% CI	PR	95% CI	PR	95% CI
**Socioeconomic status**								
High (ref.)								
Low	1.82	1.37, 2.43	1.64	1.02, 2.62	2.00	1.28, 3.12	2.07	1.02, 4.19
Medium	1.06	0.82, 1.37	1.20	0.80, 1.78	0.99	0.65, 1.51	1.41	0.70, 2.87
**Household characteristics**								
High (ref.)								
Low	1.12	0.84, 1.49	1.10	0.71, 1.70	0.98	0.62, 1.56	1.80	0.87, 3.74
Medium	1.20	0.92, 1.57	1.12	0.73, 1.70	1.10	0.72, 1.68	1.89	0.93, 3.85
**Racial & ethnic minority status**								
High (ref.)								
Low	1.04	0.75, 1.44	1.04	0.60, 1.79	1.00	0.60, 1.66	1.23	0.60, 2.52
Medium	1.03	0.80, 1.32	1.28	0.87, 1.88	0.93	0.62, 1.40	1.20	0.63, 2.26
**Housing type & transportation**								
High (ref.)								
Low	1.53	1.17, 2.02	1.51	0.97, 2.35	1.54	1.01, 2.37	1.54	0.76, 3.11
Medium	1.07	0.82, 1.39	1.02	0.68, 1.52	1.06	0.70, 1.61	1.47	0.74, 2.92
**Overall**								
High (ref.)								
Low	1.63	1.23, 2.17	1.49	0.92, 2.40	1.71	1.10, 2.67	2.14	1.05, 4.39
Medium	1.15	0.89, 1.49	1.45	0.99, 2.12	0.99	0.65, 1.52	1.81	0.90, 3.64

^a^ Each social vulnerability model controlled for crime type and incident year

^b^ Each social vulnerability model controlled for incident year

PR = Prevalence Ratio, CI = Confidence Interval

## Discussion

Using crime reports from the Prince George’s County Police Department, we quantified the prevalence of social media-involved violent crimes across social vulnerability levels. We found that lower social vulnerability was associated with a higher prevalence of social media-involved violent crimes in Prince George’s County, Maryland. We also found that young, White, male, and non-resident individuals were more likely to be victims of social media-involved violent crimes. These results provide a novel understanding of the relationships between place, social media, and violent crimes.

Although more evidence is needed, the relationship between lower social vulnerability and a higher prevalence of social-media involved violent crimes could be attributed to social media platforms catering to affluent individuals (Rideout et al. 2022). The present analysis showed a significant positive association between robberies and social media-involved crimes, consistent with prior research using this database (Garcia Whitlock et al. 2023). Social media revenue largely derives from advertising (Raffoul et al. 2023), with affluent users potentially subjected to targeted marketing for luxury goods. Given their greater access to the internet relative to less affluent individuals (Rideout et al. 2022), affluent individuals may post images and videos showcasing socially valued items (e.g., expensive clothing) to maintain high social status and popularity. Geographic details in these posts may provide sufficient information for others to commit robberies. In addition, as mentioned earlier, the social media utilization rate tends to be slightly higher among higher-income households (Gottfried 2024). For these reasons, this may be why our original hypothesis of higher social vulnerability associating with a higher prevalence of social media-involved crimes was disproven.

The current investigation yielded critical implications for public health practice and policy decision-making. Our findings support the implementation of collecting incident-based social media involvement in crime reports among law enforcement agencies across the United States and internationally. To our knowledge, this is the first study to examine social vulnerability and the prevalence of social media-involved crimes. Therefore, these insights can inform could inform various stakeholders in meaningful ways. Injury epidemiologists can include social media usage measures when studying violence risk factors and target their surveillance efforts on social media-involved crimes in lower vulnerability areas. Prevention scientists could use these findings to tailor programs differently for lower versus higher vulnerability communities, with a greater emphasis on social media safety in less vulnerable areas. Police departments could use this information to allocate resources and tailor community education efforts based on neighborhood vulnerability levels, potentially informing new training protocols for officers investigating social media-involved crimes. Community engagement could involve holding town halls to present findings and gather input on potential interventions. Injury epidemiologists, prevention scientists, and enforcement agencies could translate this study’s insights into practical, community-specific strategies to address the intersection of social vulnerability, social media use, and violent crime.

This study has limitations. Analyses are based on one US county with high median household and education levels compared to the general US population. Thus, our findings have limited generalizability to counties with different sociodemographic characteristics. Statistical inferences may be susceptible to measurement error and bias from various sources. Some crime report information is subjective, relying on officers’ perceptions in the absence of a surviving victim, leading to potential misclassification. Additionally, there are reporting limitations related to the “dark side of crime” – crimes that go unreported or undetected (Morgan and Truman 2020). Analyses from the National Crime Victimization Survey show that certain types of crimes, such as robbery, tend to be underreported compared to others, like assault (Morgan and Truman 2020). It is possible that crime underreporting is correlated to a victim’s race/ethnicities and social vulnerabilities, which may have skewed the prevalences presented in our analysis. Confounding bias is possible due to unmeasured factors affecting the relationship between social vulnerability and social media-involved violent crimes (Hernán and Robins 2020). Collider bias may affect our results, as police interactions are not randomly distributed (Goel et al. 2016; Hannon 2020; Knox et al. 2020; Knox and Mummolo 2020a, b; Pierson et al. 2020). Lastly, this study’s findings are correlational and do not establish causal relationships.

Despite these limitations, the current investigation has several strengths. We analyzed a novel measure of social media involvement in violent crimes developed by the Prince George’s County Police Department, which serves over 900,000 people (Garcia Whitlock et al. 2023). We modeled the prevalence of this measure as a function of validated social vulnerability measures at the census tract level, which could inform prevention efforts. We fit stratified models by crime type (assault and homicide, robbery, and sexual offense) in the primary and sensitivity analyses. These stratified analyses provided a thorough way to assess the relationship between social vulnerability and social media-involved crimes. We analyzed recent crime report data spanning 2018 to 2023. We characterized the application type distribution (e.g., Instagram, TikTok) among social media-involved violent crimes and examined the association between specific types of violent crimes by social media type. We employed a population-averaged model accounting for within-census tract correlation (Liang and Zeger 1986). Lastly, we provided novel insights into the role of social media in our lives beyond information sharing about products and lifestyles (Yeung 2018).

## Conclusions

Using crime report data in Prince George’s County, Maryland, we found that lower social vulnerability is associated with a higher prevalence of social media-involved violent crimes. These empirical insights underscore the need for nationally representative estimates of social media-involved crimes. Comprehensive data collection at the national and international levels provides the capacity to elucidate the relationships between neighborhoods, social media, and population health.

## Electronic supplementary material

Supplementary Material 1

## Data Availability

The dataset supporting the conclusions of this article can be obtained by emailing a request to the Prince George’s County Police Department.

## Abbreviations

aPR: Adjusted Prevalence Ratio
SES: Socioeconomic Status
