# Supplementary material for: Lower social vulnerability is associated with a higher prevalence of social media-involved violent crimes in Prince George’s County, Maryland, 2018–2023
Source: Inj Epidemiol. 2024 Sep 30;11:54. doi: 10.1186/s40621-024-00538-w (PMC11443827; doi:10.1186/s40621-024-00538-w)
Supplement: Supplementary file 1 — Supplementary Material 1 [file 40621_2024_538_MOESM1_ESM.docx]

Supplementary Materials for *“Lower Social Vulnerability is Associated with a Higher Prevalence of Social Media-Involved Violent Crimes in Prince George’s County, Maryland, 2018-2023”* by Bather et al.

**Supplemental Table 1.** Categorization of Violent and non-Violent Crimes in Prince George’s County, Maryland, 2018-2023.

| Violent Crimes | Non-Violent Crimes |
| --- | --- |
| - Aggravated Assault - Simple Assault - Robbery - Carjacking - Homicide (Murder and Nonnegligent Manslaughter) - Homicide (Negligent Manslaughter) - Sex Offense (Rape) - Sex Offense (Sexual Assault with An Object) - Sex Offense (Sodomy) - Kidnapping/Abduction | - Motor Vehicle Theft - Unauthorized Use of a Vehicle (UUV) - Homicide (Justifiable) - Burglary/Breaking and Entering - Stolen Property Offenses - Disorderly Conduct - Driving Under Influence - Drunkenness - Non-Specified Must Appear Traffic Offenses - Intimidation (Threats) - Threat (Arson) - Sex Offense (Fondling) - Sex Offense (Incest) - Sex Offense (Statutory Rape) - Theft (Bicycles) - Theft (From Building) - Theft (From Motor Vehicle) - Theft (of Services) - Theft (Of Services) - Theft (other) - Theft (Other) - Theft (Parts/Accessories from Motor Vehicle) - Theft (Purse-snatching) - Theft (Shoplifting) - Embezzlement - Trespassing - Fraud (Credit Card/Automated Teller Machine) - Fraud (False Pretenses/Swindle/Confidence Game) - Fraud (Wire) - UUV (Unauthorized Use of Vehicle) - Arson - Counterfeiting/Forgery - Drug Equipment Violations - Drug/Narcotic Violation - Extortion/Blackmail - Human Trafficking (Commercial Sex Acts) - Human Trafficking (Involuntary Servitude) - Pornography/Obscene Material - Prostitution - Prostitution (Assisting or Promoting) - Prostitution (Purchasing) - Telephone Misuse - Violation of No Contact/Protection Orders - Weapon Law Violations - Destruction/Damage/Vandalism Property |

**Supplemental Table 2.** Results from the negative binomial regression models of social vulnerability and social media-involved violent crimes in Prince George’s County, Maryland, 2018-2023.

|  | **All crime types^a^** | | **Crime type (Subgroup analyses)^b^** | | | | | |
| --- | --- | --- | --- | --- | --- | --- | --- | --- |
|  |  | | *Assault & Homicide* | | *Robbery* | | *Sexual Offense* | |
|  | *PR* | *95% CI* | *PR* | *95% CI* | *PR* | *95% CI* | *PR* | *95% CI* |
| **Socioeconomic Status** |  |  |  |  |  |  |  |  |
| High (ref.) |  |  |  |  |  |  |  |  |
| Low | 1.83 | 1.36, 2.44 | 1.64 | 1.02, 2.63 | 2.00 | 1.26, 3.16 | 2.08 | 1.01, 4.31 |
| Medium | 1.06 | 0.81, 1.38 | 1.20 | 0.80, 1.78 | 0.99 | 0.64, 1.53 | 1.41 | 0.68, 2.90 |
|  |  |  |  |  |  |  |  |  |
| **Household Characteristics** |  |  |  |  |  |  |  |  |
| High (ref.) |  |  |  |  |  |  |  |  |
| Low | 1.12 | 0.83, 1.50 | 1.10 | 0.70, 1.71 | 0.98 | 0.61, 1.57 | 1.79 | 0.85, 3.79 |
| Medium | 1.20 | 0.91, 1.57 | 1.12 | 0.73, 1.70 | 1.10 | 0.72, 1.69 | 1.88 | 0.91, 3.91 |
|  |  |  |  |  |  |  |  |  |
| **Racial & Ethnic Minority Status** |  |  |  |  |  |  |  |  |
| High (ref.) |  |  |  |  |  |  |  |  |
| Low | 1.04 | 0.75, 1.45 | 1.04 | 0.60, 1.80 | 1.00 | 0.59, 1.68 | 1.23 | 0.58, 2.59 |
| Medium | 1.03 | 0.79, 1.33 | 1.28 | 0.87, 1.88 | 0.93 | 0.61, 1.41 | 1.20 | 0.62, 2.31 |
|  |  |  |  |  |  |  |  |  |
| **Housing Type & Transportation** |  |  |  |  |  |  |  |  |
| High (ref.) |  |  |  |  |  |  |  |  |
| Low | 1.54 | 1.16, 2.03 | 1.51 | 0.97, 2.36 | 1.55 | 1.00, 2.40 | 1.55 | 0.75, 3.20 |
| Medium | 1.07 | 0.82, 1.40 | 1.02 | 0.68, 1.53 | 1.06 | 0.70, 1.63 | 1.46 | 0.72, 2.98 |
|  |  |  |  |  |  |  |  |  |
| **Overall** |  |  |  |  |  |  |  |  |
| High (ref.) |  |  |  |  |  |  |  |  |
| Low | 1.63 | 1.22, 2.19 | 1.49 | 0.92, 2.41 | 1.71 | 1.09, 2.70 | 2.15 | 1.03, 4.50 |
| Medium | 1.15 | 0.89, 1.49 | 1.45 | 0.98, 2.13 | 1.00 | 0.65, 1.53 | 1.81 | 0.88, 3.70 |
| PR = Prevalence Ratio, CI = Confidence Interval | | | | | | | | |
| **^a^** Each social vulnerability model controlled for crime type and incident year | | | | | | | | |
| **^b^** Each social vulnerability model controlled for incident year | | | | | | | | |
